# The importance, consequences and treatment of psychosocial risk factors in heart disease: less conversation, more action!

**DOI:** 10.1007/s12471-023-01831-x

**Published:** 2023-11-28

**Authors:** Nina Kupper, Sophie van den Houdt, Petra M. J. C. Kuijpers, Jos Widdershoven

**Affiliations:** 1 Center of Research on Psychological Disorders and Somatic Diseases, Department of Medical and Clinical Psychology, Tilburg University, Tilburg, The Netherlands; 2 Hart voor de Zaak, Cardiopsychiatric Consultancy, Maastricht, The Netherlands; 3 Department of Cardiology, Elisabeth-TweeSteden Hospital, Tilburg, The Netherlands

**Keywords:** Psychosocial risk factors, Cardiac rehabilitation, Screening, Treatment, Recommendations

## Abstract

Psychosocial factors play a significant role in the incidence and prognosis of cardiovascular disease with a rapidly increasing body of knowledge, as acknowledged by their inclusion in the European Society of Cardiology cardiovascular prevention guideline since 2012. Nevertheless, psychosocial risk is not consistently assessed, acknowledged and treated in daily clinical practice. Therefore, adopting a multidimensional approach that encompasses biological, psychological, and social factors is crucial for understanding the dynamic nature of cardiovascular health and disease, delivering patient-centred care, and developing effective interventions to ultimately enhance health and satisfaction with contemporary medicine and care. The current review summarises the state-of-the-art evidence for screening and treating psychological risk factors in coronary heart disease, heart failure, and atrial fibrillation in the context of cardiac rehabilitation, along with accompanying recommendations. The limited adoption of routine screening, despite longstanding recommendations, highlights the importance of prioritising the implementation and expansion of routine screening in primary and secondary prevention. To advance psychosocial treatment, a standardised and personalised approach including comprehensive education, physical exercise, and psychosocial support with a focus on patient-reported outcomes is crucial. Treating heart and mind together has the potential to decrease psychosocial risk while enhancing the prognosis and quality of life, therefore delivering true patient-centred care.

## Introduction

By arguing a mind-body dualism, Descartes demythologised the body and paved the way for three centuries of progress in medicine, shaping the biomedical model of health and disease. Simultaneously, by isolating the mind, dualism denied its significance in individuals’ experiences of health [1]. More directly, it denied the option that, for example, personality, emotions, and behaviours could affect physiology, and vice versa. When, in 1947, the World Health Organisation defined health as a state of complete physical, mental, and social well-being, a new understanding of health emerged, challenging the hegemony of biomedicine. A multidimensional approach, including biological, psychological, and social factors, is essential to understand the dynamic nature of cardiovascular health and disease, to practice patient-centred care and to develop effective interventions, ultimately increasing health and satisfaction with contemporary medicine and care [1].

The current paper embraces this multidimensional approach and summarises extant literature linking psychosocial risk factors with common cardiovascular conditions: coronary heart disease (CHD), heart failure (HF), and atrial fibrillation (AF). We also provide a state-of-the-art overview of psychosocial screening and treatment, in the context of cardiac rehabilitation (CR). The rationale for the current endeavour is that there are multiple compelling, evidence-laced reasons for cardiologists to be skilled in recognising and managing psychosocial risk (Tab. 1, right panel). In addition to reiterating the current state of the art [2], our paper is a call to action for clinicians working in cardiology and ends with concrete recommendations.

**Table 1:** Overview of psychosocial risk factors (left) and their link with cardiac practice (right)

Psychosocial risk factors		Link with cardiac practice [3]
Emotional factors	Stress-related problems	
*Depression* *Anxiety* *Anger* *Hostility (and TABP)* *Acute severe emotional reactivity*	*Work stress* *Financial stress* *Social stress* *Distressed personality (Type D)* *Vital exhaustion* *Life circumstances (early life, traumatic events)* *Socio-economic position*	– Significant risk factor for CHD, HF, AF, AP – Highly prevalent in cardiac practice – May trigger cardiac events – Linked to behavioural and cardiovascular risk factors – Forms a barrier to medical interventions and cardiac rehabilitation – Impacts patients’ quality of life and well-being – Commonly masquerades as cardiac symptoms

*TABP* Type A behaviour pattern, *CHD* coronary heart disease, *HF* heart failure, *AF* atrial fibrillation, *AP* angina pectoris

## Psychosocial risk defined

**Fig. 1:**
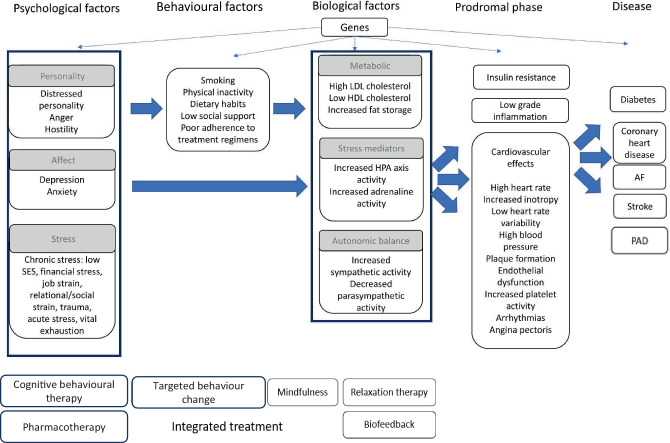
Plausible pathways from psychological risk factors to heart disease and options for treatment. *SES* socioeconomic status, *LDL* low-density lipoprotein, *HDL* high-density lipoprotein, *AF* atrial fibrillation, *PAD* peripheral artery disease

Psychosocial risk factors for heart disease may be divided into two categories: emotional factors and stress-related factors (Tab. 1, left panel; [3]). Patients with emotional problems will show symptoms of anxiety, depression, anger, and hostility, or are overwhelmed with acute severe emotions (e.g., causing Takotsubo or stress cardiomyopathy) or develop post-traumatic stress disorder (PTSD) after the event. Patients with stress-related problems though will report strain in the context of work, relationships, life circumstances (early life events, trauma, lack of social support), have a generalised distressed disposition or poor socioeconomic position. Psychosocial risk factors tend to cluster within patients in a tightly knit network of behavioural and cardiovascular risk factors [4], and recent research has identified specific within-person profiles [5]. Figure 1 illustrates how psychosocial risk factors affect cardiovascular risk factors and illness through behavioural and biological pathways.

## Link with prognosis and quality of life

### Coronary heart disease

Multiple psychosocial factors have been identified as highly relevant for CHD. This is evident by the attention received from the American Heart Association and the European Society of Cardiology (ESC), the latter including the advice to assess and treat these risk factors in the ESC Cardiovascular prevention guideline since 2012 [6]. In brief, not only depression and anxiety [7], but also lack of social support and intimate relationships [8], socioeconomic status [9], chronic stress and trauma/PTSD [10], anger [11], hostility [12], Type D (distressed) personality [13] and vital exhaustion [14] have shown a robust relationship with an increased risk of major adverse cardiac events and mortality from CHD. Importantly, *all* psychosocial risk factors are detrimental to patients’ well-being and quality of life (QoL)(e.g., [15]).

### Heart failure

Depression and anxiety are common in HF and are associated with a poor prognosis, increased hospitalisation and mortality risk, likely mediated by both behavioural and biological processes [16]. Psychosocial risk factors relevant in CHD keep exerting their detrimental influence when the disease has progressed into HF. In patients with HF, the negative impact of receiving and wearing an internal cardiac defibrillator on emotional stress and QoL is high [17]. While some of these symptoms recover, others (e.g., heart-focused anxiety and depression) often do not improve on their own and may benefit from psychological treatment and CR [18]. The 2021 ESC guideline on diagnosis and treatment of acute and chronic HF recommends assessment and treatment for depression [19].

### Atrial fibrillation

It is only quite recently (during the last decade) that psychosocial factors have become more prominent in AF research, in the early years starting with health-related quality of life (HRQoL). Research in patients with AF has shown a significant role for depression, anxiety, anger, stress, panic disorder, PTSD, Type D personality, socioeconomic status and social support. A recent meta-analysis demonstrated that anxiety, depression, anger, and work stress all increase the incidence of AF [20]. Also, psychological trauma [21] and anxiety after cardiac surgery [22] have been implicated in increasing AF risk. In patients with AF, emotional and stress-related problems are more prevalent than in the general population, impacting patients’ HRQoL, symptom burden and increasing the risk of adverse events, such as stroke and major bleeding [23, 24]. The 2020 ESC guideline on atrial fibrillation summarised that AF patients often develop anxiety, depression and poorer HRQoL when having a Type D personality [25]. Based on this evidence, it recommended integrated patient-centred management, including psychosocial assessment and treatment. This contrasts sharply with current clinical practice, where the focus is on physical symptoms and the main discussion is on rhythm or rate control.

## Screening for psychosocial risk factors

Screening for psychosocial risk prior to CR is becoming increasingly common [26] and is recommended upon entering CR [27]. Throughout the Western world, this has been advocated in scientific statements (e.g., [2]) and by secondary prevention guidelines (e.g., [28]), as this may not only be indicative of whether an evaluation by a psychologist or psychiatrist is advisable, but screening could also identify a patient’s unique needs [27] and lay bare the barriers that need to be overcome to successfully change health behaviour. Consequently, it is advised in current Dutch CR guidelines that a mental health professional helps decide which level of psychosocial care is needed [29]. Furthermore, patients should also be screened upon completion of CR with follow-up assessments at six and twelve months. However, in practice this recommended protocol is not consistently followed [26].

One reason why screening is limited pertains to the feasibility of screening [30]. Current methods that are deemed as reliable screening practices for the assessment of psychosocial risk are standardised structured interviews [26] and validated questionnaires [31]. Screening interviews may increase the opportunity to provide more context surrounding psychosocial risk [31]. However, they are highly unpractical in cardiological practice: too time-consuming, expensive and require knowledge on when and how to take further action (e.g., referring to a psychologist), which is often lacking. As for self-reported screening assessments, the process often involves utilising full-scale instruments that focus on assessing a single risk factor. In 2015, a position paper by the ESC CR Section listed psychometric assessment options for each of the psychosocial risk factors [32]. However, combining multiple scales results in a lengthy screening process. To improve the feasibility of screening, we need a comprehensive, quick and stepped screening approach to evaluate psychosocial risk factors simultaneously, adding a clinical interview with a psychologist when necessary.

Psychosocial screening currently predominantly focuses on depression and generalised anxiety [26, 30], which is in contrast with ESC guidelines [6, 28] recommending to assess and treat the set of risk factors reviewed above. Screening for this broader set should be followed up by a more extensive care pathway. For this to crystallise, research into the effectiveness of screening and risk factor specific effects of psychological treatment on medical outcomes (morbidity and QoL) is needed. A first step was recently taken. We validated a comprehensive psychosocial screening instrument that includes all psychological risk factors recommended to screen for among the Dutch general population, Dutch CHD patients and German coronary angiography patients (e.g. [33]). The one-page instrument quickly and reliably screened for psychosocial risk factors and can serve as a risk triage in a stepped care approach within the realms of CR and medical psychological care [33].

Several recommendations arise for the screening phase, pertaining to the screening instrument, care pathway and eligibility considerations. In particular, echoing recent insights in CR, psychosocial screening prior to CR should be done in all cardiac patients. There is sufficient evidence that patients with AF, HF and non-obstructive CHD are in need of psychosocial help. In addition, to truly be effective in treating psychosocial risk, we should also expand routine psychosocial screening in cardiac outpatient and primary care practices, whereas in the Netherlands it now only takes place in the context of CR, predominantly tailored at acute and chronic coronary syndrome patients. An evidence-based harmonisation effort should take place with respect to the instruments, timing and risk factors assessed. Research is needed to examine the clinical effectiveness and cost-effectiveness of comprehensive screening for psychosocial risk. Finally, screening instruments should be made to fit people with reduced health literacy, poorer language understanding and lower digital skills (Tab. 2).

**Table 2:** Recommendations for psychosocial screening in cardiac patients

Screening
Use a comprehensive, quick and stepped screening approach
Include all cardiological diagnoses to screen for psychosocial risk
Harmonise instruments, timing, eligibility and procedures across hospitals
Routinely screen for psychosocial risk as a primary prevention measure
Improve health literacy and adapt screening, psycho-education and PEP instructions and digital design, so that they are widely comprehensible and accessible
Improve oversight on adherence to clinical guidelines
Do research into the clinical effectiveness and cost-effectiveness of comprehensive screening for psychosocial risk

*PEP* Psycho-educational prevention

## Psychosocial treatment in the context of cardiac rehabilitation

CR is a fundamental strategy in the further prevention after a cardiovascular event, and carries a class IA recommendation in international guidelines for improving outcome after an acute coronary event or revascularisation. Across centres and countries, CR differs enormously in terms of the eligible population and CR methods. A recent meta-regression analysis demonstrated that exercise-based CR provides important benefits to patients following ACS or revascularisation in terms of morbidity, mortality, hospitalisation, costs and HRQoL [34].

Stepped care approaches are becoming increasingly popular in CR (e.g., [35]), as they effectively differentiate patients’ individual needs based on risk assessment. By aligning care delivery with the specific needs and treatment responsiveness of each patient, stepped care ensures a patient-centred approach. Regular monitoring and evaluation of treatment outcomes allow for adjustments to be made. This approach ultimately prevents overtreatment of patients who require less care and undertreatment of those in need of more psychosocial attention [35].

### Current situation

The Psycho-Educational-Prevention (PEP) module of CR in the Netherlands is an example of a stepped care approach, which aims to enhance behavioural lifestyle changes and alleviate psychosocial distress to minimise the risk of recurring events. The PEP module targets emotional disbalance by offering sessions in stress management, relaxation training, emotion regulation (anxiety, depression, anger), social relationships and perfectionism in addition to the physical CR. This allows for tailoring based on patients’ needs and risk profiles and adapting it to hospitals’ preferences and resources. Consequently, this has led to a large heterogeneity in the number of sessions offered, duration of treatment, required presence of the partner and content-wise deviance from the PEP guideline.

### Evidence-base

In general, meta-analytic evidence shows that psycho-educational programmes (health education and stress management), one of the core elements of CR, reduce both morbidity and mortality in patients with CHD, as well as improving mood [36]. Delivering patient education rendered a 60% reduction in clinical anxiety and a 35% reduction in clinical depression compared with care as usual [37]. CR often includes various psychological interventions for lifestyle change or distress management. However, the additional benefit of specific psychological interventions on depression, anxiety, QoL, cardiac morbidity and mortality is not well investigated and considerable uncertainty remains about under which conditions these interventions exert their optimal effects [38]. The CR-PEP module suffers from a lack of a clear consensus definition of what psychosocial intervention is and lacks national and international consistency in design and delivery [39]. As this complicates effect studies, a more precise definition, renaming and demarcation of the effects sought will bring this field forward.

The 2011 Dutch multidisciplinary guideline for CR [29] recommends treating depression and anxiety symptoms. The guideline recognises that there are more relevant psychosocial risk factors besides depression and anxiety, but as evidence for treatment effects on morbidity and mortality was still lacking, advice was withheld. Below, we summarise the current evidence for treatment of anxiety and depression first, and then move on to discuss some of the other psychological risk factors, and provide recommendations in Tab. 3.

**Table 3:** Recommendations for advancing psychosocial treatment in cardiac patients in CR

Treatment
Focus on HRQoL as an outcome measure for clinical trials on psychological interventions, not on cardiac morbidity and mortality
Define, design and deliver psychological, social or educational treatment in a more precise and standardised way
Make the PEP module more versatile and offer patient-tailored treatment programmes addressing the risk profile of the patient
Make use of digital health options, such as m‑health or web-based care, as an add-on and never as replacement of a mental health care professional

*HRQoL* health-related quality of life, *PEP* psycho-educational prevention

### Treatment of depression & anxiety

Depression and anxiety in recovering CHD patients negatively affects outpatient completion rates of CR [40]. While meta-analyses show that cognitive behavioural therapy (CBT) in cardiac patients is effective in reducing depression and anxiety [41], therapeutic interventions do not seem to affect mortality that much. Importantly, studies are generally not powered well enough to find mortality effects, certainly not in the current era of declining mortality rates. Pharmaceutical trials show effectiveness of selective serotonin reuptake inhibitors (SSRIs) and benzodiazepines. Tricyclic antidepressants should not be used in cardiac patients. For recommendations see Kahl et al. [42]. Patients with a low QoL have higher morbidity rates. CR improves QoL and a better QoL baseline score leads to fewer dropouts in CR [32]. Only one study to date has examined the effect of improvement in depression on mortality in the context of CR and found that a reduction in depression was associated with a substantial reduction of mortality, comparable with non-depressed cardiac patients [43]. In HF, psycho-education was shown to reduce HF symptoms while improving depressive symptoms, and lessons from SADHART-CHF and MOOD-HF teach us that SSRIs inconsistently improve mood, hospitalisation rates, or mortality risk in HF, with CBT and exercise performing superiorly [44]. All these effects were in face-to-face CBT. In-person contact could be supplemented by e‑health and m‑health features, such as smartwatches and treatment apps, but can never be completely replaced.

### Treatment of chronic stress

It is important to identify the stressors that are relevant to the individual patient. Besides the effect of stress on patients’ well-being, stress may pose a barrier for successful CR and changing health behaviour. Therefore, stress management is recommended as a first-line intervention and may also serve as a crucial prerequisite for successful CR [32]. A systematic review and meta-analysis suggested that stress management interventions may actively encourage health behaviours, contribute to an improved prognosis and were demonstrated to improve QoL [45]. Moreover, the SUPRIM study showed that in the context of traditional CR, patients in the +CBT group (focusing on stress management) had a 41% lower rate of recurrent CVD events (fatal and non-fatal) and 45% fewer recurrent events [46].

Recently, there has been a growing emphasis on the utilisation of meditation interventions for stress management in order to decrease cardiac risk [2]. This approach serves as an alternative to conventional medical interventions, primarily due to its advantageous health effects and cost-effectiveness. Meditation is suggested to hold potential benefits in patients with established CHD, although the evidence supporting this claim is rather modest [2].

### Treatment of anger and hostility

The combination of motivational interviewing and CBT was found to be successful in reducing anger levels in cardiac patients [47]. An early study further showed that a psycho-educational CR programme combined with exercise was successful in reducing hostility, and psychological distress in general, and improving QoL [48]. Besides these and a few other examples, there is a scarcity of research focusing on efficacious interventions for reducing the detrimental effects of anger and hostility.

### Quality of life

As treatment of patients with CHD is aimed at improving HRQoL, in addition to the physical status, the inclusion of HRQoL as a primary outcome measure in clinical trials as well as routine monitoring of HRQoL in cardiac practice is warranted. The 14-item HeartQoL questionnaire is a CHD-specific tool which validly assesses HRQoL in the physical and emotional domain and for which European (EUROASPIRE) comparison data are available [49]. For AF, several AF-specific HRQoL measures have been developed, the AFEQT being the most favourable methodologically [50]. For HF, disease-specific instruments such as the Minnesota Living with Heart Failure Questionnaire (MLHF) and the Kansas City Cardiomyopathy questionnaire (KCCQ) are valid multidimensional options, comprising physical, mental, social, environmental and role functioning.

## An agenda for the future: research and practice

Future research on psychological risk factors in the context of cardiology should focus on routine screening in outpatient and primary care practices and broadening the screening scope in CR. Implementation studies should take heed of research on barriers and facilitators and design effective care pathways. In addition, the scope with respect to social determinants of heart disease needs to be broadened as well to include, for example, sex and gender, race/ethnicity, financial resources, health literacy and the environmental context ((rural vs. residential vs. inner city) in terms of accessibility of care, driving distance, level of air pollution, and heat exposure). In terms of screening, large heterogeneity exists in which psychosocial risk is screened for and which instruments are used to screen. It is essential to reach consensus about screening instruments. To date, no clinical trial has tested the efficacy of the PEP module in CR, one of the reasons being that its heterogeneity makes comparison difficult, if not futile. There is evidence though (reviewed above) for individual treatments that may be extrapolated to the PEP/CR context. For psychological treatment within the context of CR to have more impact and a broader uptake, an adaptive clinical trial that seeks to identify the most effective and clearly defined intervention and tools among a wide variety of possible strategies would be recommended. In such a trial, treatment should be tailored to the individual patient’s risk profile, and the outcome assessment should predominantly be focused on patient-reported outcomes. The current review provides some recommendations for screening and treatment (see Tab. 1, 2 and 3).

**Fig. 2:**
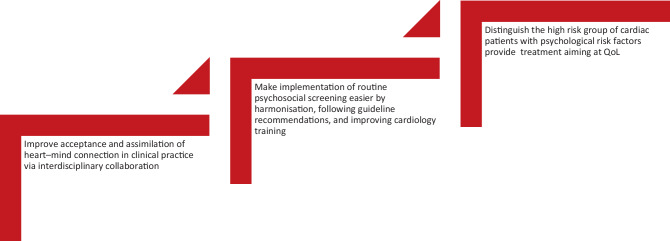
Clinical practice recommendations

Despite the evidence and increased knowledge of the relationships and mechanisms between psychosocial risk factors and heart disease, the inclusion in international guidelines, and despite thorough dissemination of this research in top-tier medical journals, the science of heart-mind interaction has only limitedly been assimilated in cardiac clinical practice. There may be several reasons for this, an important one pertaining to physician/cardiologist educational programmes, which are lacking multidisciplinary fo﻿cus; another pertaining to the fact that most practising clinicians are trained and operate under models of care that emphasise biomedical and physiological risk factors. It will be easier to acknowledge the presence, suffering, and impact of psychosocial risk factors in your patients by routinely screening for those factors (Fig. 2).

## Conclusion

In conclusion, psychosocial risk factors have a large clinical relevance for cardiological practice. International and national guidelines include psychological risk factors and recommend assessing and treating these factors. Nevertheless, implementation of guideline recommendations trails behind, high-quality studies on the additional effectiveness of psychological treatment in CR are lacking due to a lack of clarity in treatment definition and delivery, and cardiologists are still being trained under dualistic models of care, lacking knowledge and insight on psychosocial risk. We need improvements in all these aspects, to treat the heart and the mind together, and to deliver true patient-centred care.
